# Am. Med. Ass. Letter from Senior Editor

**Published:** 1857-06

**Authors:** 


### Letter from Sen. Editor.

Nashville, Tenn., May 1857.

Prof. Marsh — *Dear Sir* : Upon leaving Keokuk we proceeded on the steamer Die Vernon, which unfortunately in the night, when the surface of the water was enveloped in a dense dark fog, ran upon a sand bar above the city of Alton, where we remained, notwithstanding the most vigorous efforts to its removal, for twenty-four hours. At St. Louis we took a Louisville boat, and at Smithland, Ky., at the mouth of the Cumberland river, we embarked on board a Nashville packet and in time arrived at the city where we were provided with comfortable quarters. We met at Smithland some twenty or more of our medical brethren, among whom was the venerable President of the Association, Dr. Pitcher, of Detroit. We journeyed together and the time flew most pleasantly as the boat plied rapidly the narrow but beautiful river.

The Association met in the new and splendid State House, where we found a good delegation from almost all parts of the Union ; and the proceedings did not lack so much their usual interest as might have been expected, from the fact, which was quite too general, that the standing committees were defective in their reports.

A discussion arose, which, because the subject, was one of great interest. We allude to the standard of medical education, the claims of representatives on the part of institutions, and the conduct of members admitted into fellowship within the Association. During the discussion it was plain that the Association will purge itself of all charlatanism whenever the facts are presented for their action. It was admitted, that although the domestic rights of the several schools should not be interfered with, yet no school will receive its sanction or endorsement, unless it comes up to the general requirements and long established usages.

Dr. Eve was elected President, unanimously, and made an able presiding officer. The physicians and citizens of the city provided rich banquets in honor of the profession assembled. and as these parties and assemblies were liberally attended by the beautiful and interesting ladies of the city, attired in their costly and splendid robes, they were very interesting to look upon by a stranger visitor. The ladies were, as they always are, objects of interest to the members, because of their bright smiles, intelligent and expressive countenances ; and doubtless a little acquaintance with them would have confirmed the favorable speculations as to the possession of head and heart.

The widow of the late President Polk, an accomplished and excellent lady, received the members of the Association, in accordance with her generous invitation, in a most cordial and hospitable manner, and we are in justice constrained to say that this visit was one of the highest gratifications we enjoyed during our stay.

But we are opposed, and ever have been since the organization of the Association, to these great displays and festive throngs. They are inconsistent with the objects of the Association and with the grand pursuits of its members. We meet for different objects and from different motives ; but permit this practice to go on in the future, and the Association will gather together those who can find more enjoyment in balls, routs, and assemblies than in the cultivation of the mind by the interchange of opinion and sentiment. How much better would the objects and designs of the Association be met and answered, if at the close of its deliberations, the members would organize into a sort of an *ex-cathedra* meeting, for the purpose of cultivating a more social feeling, and of giving to each other their experience of the diseases with which they are required to contend in their several localities, difficulties they are compelled to meet, and overcome, and the character of the opposition to regular medicine. This too would be a favorable opportunity of exposing the conduct of demagogues and charlatans who cling for character and standing to regular medicine, but whose empirical proceedings and immoral conduct bring opprobrium upon it. These should be pointed out and exposed to the gaze of the profession, in order that it may be purged from such irregularities and licentiousness. Unless this be more effectually done, and the pruning hook laid at the root of these rank weeds, everywhere choking and strangling regular medicine, until it is seen to sicken wherever they grow, the objects of the Association will not be fully accomplished. How much more profitable such a mode of procedure, than to engage in a grand banquet where all cannot engage, but many must stand back and gaze in silence, while others are in the midst of hilarity and glee, and in the highest enjoyment. The membership of the Association is made up of the old and the young, the grave and the gay ; a few can "trip the light fantastic toe," while others cannot define the difference between a cotillon and a contra dance ; and then others are conscientiously opposed to that mode of amusement. Such is the composition of the membership, and therefore, the benefits, if we may so term these banquets, are not equally distributed. According to our plan all could engage, or if not actually, would be deeply interested listeners. We trust therefore, that a former expression of sentiment touching these grand displays will be rigidly adhered to in order to the preservation and the further elevation of the true dignity of a body of learned and scientific men. If the city in which the Association meets, would expend their means in aid of those who are at great sacrifice of time and money in attendance upon these conventions, either by reducing the fares on the railroads, the purchase of books, or the appliances in which there would be substantial benefit conferred upon a class of men who are less able to endure these expenses than the members of any of the other liberal professions, a more enduring benefit would be conferred and a deeper gratitude felt and remembered.

We had the pleasure of meeting many old friends and distinguished individuals of the profession among whom was Prof. Dungleson, Dr. Wister, both of Philadelphia. Professors March, Hooker, Palmer, Green, Breckinridge, with many others.

But to the proceedings, an abstract of which I send you.

